# Fire severity as a key determinant of aboveground and belowground biological community recovery in managed even‐aged boreal forests

**DOI:** 10.1002/ece3.10086

**Published:** 2023-05-17

**Authors:** Leticia Pérez‐Izquierdo, Jan Bengtsson, Karina E. Clemmensen, Gustaf Granath, Michael J. Gundale, Theresa S. Ibáñez, Björn D. Lindahl, Joachim Strengbom, Astrid Taylor, Maria Viketoft, David A. Wardle, Marie‐Charlotte Nilsson

**Affiliations:** ^1^ Department of Soil and Environment Swedish University of Agricultural Sciences Uppsala Sweden; ^2^ Department of Ecology Swedish University of Agricultural Sciences Uppsala Sweden; ^3^ Department of Forest Mycology and Plant Pathology Uppsala BioCenter Swedish University of Agricultural Sciences Uppsala Sweden; ^4^ Department of Ecology and Genetics Uppsala University Uppsala Sweden; ^5^ Department of Forest Ecology and Management Swedish University of Agricultural Sciences Umeå Sweden; ^6^ Department of Wildlife Fish and Environmental Studies Swedish University of Agricultural Sciences Umeå Sweden; ^7^ Department of Ecology and Environmental Science Umeå University Umeå Sweden

**Keywords:** Boreal forest, climate change, ecosystem recovery, even‐aged forestry, fire severity, ground fire, *Pinus sylvestris*, soil biota, stand‐replacing fire

## Abstract

1. Changes in fire regime of boreal forests in response to climate warming are expected to impact postfire recovery. However, quantitative data on how managed forests sustain and recover from recent fire disturbance are limited.
2. Two years after a large wildfire in managed even‐aged boreal forests in Sweden, we investigated how recovery of aboveground and belowground communities, that is, understory vegetation and soil microbial and faunal communities, responded to variation in the severity of soil (i.e., consumption of soil organic matter) and canopy fires (i.e., tree mortality).
3. While fire overall enhanced diversity of understory vegetation through colonization of fire adapted plant species, it reduced the abundance and diversity of soil biota. We observed contrasting effects of tree‐ and soil‐related fire severity on survival and recovery of understory vegetation and soil biological communities. Severe fires that killed overstory *Pinus sylvestris* promoted a successional stage dominated by the mosses *Ceratodon purpureus* and *Polytrichum juniperinum*, but reduced regeneration of tree seedlings and disfavored the ericaceous dwarf‐shrub *Vaccinium vitis‐idaea* and the grass *Deschampsia flexuosa*. Moreover, high tree mortality from fire reduced fungal biomass and changed fungal community composition, in particular that of ectomycorrhizal fungi, and reduced the fungivorous soil Oribatida. In contrast, soil‐related fire severity had little impact on vegetation composition, fungal communities, and soil animals. Bacterial communities responded to both tree‐ and soil‐related fire severity.
4. *Synthesis*: Our results 2 years postfire suggest that a change in fire regime from a historically low‐severity ground fire regime, with fires that mainly burns into the soil organic layer, to a stand‐replacing fire regime with a high degree of tree mortality, as may be expected with climate change, is likely to impact the short‐term recovery of stand structure and above‐ and belowground species composition of even‐aged *P. sylvestris* boreal forests.

## Graphical abstract

Our results suggest that a change in fire regime from a historically low‐severity ground fire regime to a stand‐replacing fire regime as may be expected with climate change, is likely to impact the short‐term recovery of stand structure and above‐ and belowground species composition of even‐aged *P. sylvestris* boreal forests.

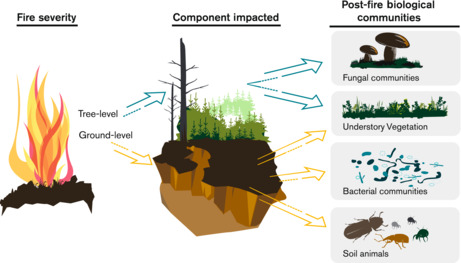

## 1 INTRODUCTION

In boreal forests, wildfires are ecologically and evolutionarily important natural agents of disturbance (Bonan & Shugart, 1989) and primary drivers of community structure, biodiversity, and ecosystem productivity (Mack et al., 2008; Turner, 2010; Wardle et al., 2012). Historically, the fire regime of boreal forests in northern Europe was characterized by low‐intensity ground fires, while intense stand‐replacing fires were relatively rare (Drobyshev et al., 2015; Granström & Niklasson, 2008; Rogers et al., 2015). However, climate warming is expected to promote changes in fire frequency, intensity and severity in boreal forests, and more stand‐replacing fires are expected in the future (Burrell et al., 2022; Kelly et al., 2013; Wotton et al., 2017), with potential consequences for ecosystem resilience and postfire recovery. Given that the boreal forest stores approximately 30% of the Earth's total terrestrial C and N stocks (Anderson, 1991; Goodale et al., 2002) and play a key role in the global C and N cycles, it is important to understand how boreal forest communities respond to changes in fire regime caused by climate warming (Granath et al., 2021; Kasischke & Stocks, 2000; Rogers et al., 2015). While short‐term fire impacts on plant communities have been described for boreal forests, few studies have addressed how tree‐related fire severity (i.e., tree mortality) impact postfire recovery aboveground and belowground relative to soil‐related fire severity (i.e., consumption of soil organic matter).

In the boreal forests of northern Europe, mature *Pinus sylvestris* (Scots pine) usually survives low‐intensity ground fires due to its thick bark and high crown base (Kuuluvainen et al., 2002), while *Picea abies* (Norway spruce) often experiences high mortality (Kuuluvainen & Aakala, 2011; Zackrisson, 1977). Many dominant understory plants, such as the ericaceous dwarf shrubs *Vaccinium myrtillus*, *V. vitis‐idaea*, and *Calluna vulgaris*, are capable of resprouting from buried meristems or rhizomes in upper soil horizons or emerge from the seed bank after fire (Schimmel & Granström, 1996). Bryophytes that dominate in late successional states (e.g., *Pleurozium schreberi* and *Hylocomium splendens*) are highly sensitive to fire (DeLuca et al., 2002), and need to recolonize with spores or vegetatively from unburnt patches. Postfire changes in forest floor conditions, such as the combustion of the organic horizon and altered physical and chemical characteristics, can influence the establishment of pioneering mosses and plants (e.g., *Polytrichum* spp., *Senecio sylvaticus*, *Betula* spp., and *Populus* sp.), but stochastic colonization is also important (Gustafsson et al., 2021; Hellberg et al., 2003; Neary et al., 1999).

The intensity of fire in boreal forests can, due to heat‐induced mortality, also affect soil microbial communities and associated ecosystem functions, thereby inflicting structural and chemical modifications of the forest floor (Day et al., 2019; Dooley & Treseder, 2011; Whitman et al., 2019). Fires resulting in extensive consumption of the soil organic horizon commonly reduce ectomycorrhizal fungal biomass, abundance, and diversity (Dooley & Treseder, 2011; Holden et al., 2016; Sun et al., 2015), particularly when tree mortality is also high (Pérez‐Izquierdo et al., 2021). These changes may persist for several decades (Bennett et al., 2017; Köster et al., 2021). Further, high‐severity fires have been shown to induce a short‐term shift in fungal community composition toward dominance of free‐living saprotrophic fungi, whereas bacterial communities appear to be more fire resistant (Bååth et al., 1995; Sun et al., 2015, 2016; Whitman et al., 2019). Changes in soil biota are often attributed to direct effects of fire on soil properties (e.g., higher pH and soil temperature) (Whitman et al., 2019), but may also be impacted by loss of vegetation (Pérez‐Izquierdo et al., 2021) and postfire succession (Adkins et al., 2020). While many studies have investigated soil microbial responses to fire, we have limited understanding of how soil microbial communities respond to variation in fire severity, and thus of how microbial processes may be affected by future changes in fire severity (Dooley & Treseder, 2011; Whitman et al., 2019).

Fire may also impact mortality rates and postfire colonization of soil fauna in boreal forest, through habitat modifications (Whitford et al., 2014; Zaitsev et al., 2016). Few studies have investigated how variation in fire severity impacts populations and recovery of soil invertebrates in boreal forests, although the depth of burn of soils has been suggested as an important determinant of their survival (Buckingham et al., 2015; Malmström, 2010; Wikars & Schimmel, 2001). This may particularly apply to soil macrofauna and mesofauna, as they should be able to escape less severe fires by moving downward within the organic layer (Janion‐Scheepers et al., 2016; Wikars & Schimmel, 2001). However, under more severe fires with high losses of the soil organic layer, soil animals lose a substantial part of their habitat including their food resources, which likely leads to reduced population densities, with potential implications for nutrient and carbon dynamics and ecosystem productivity (Gongalsky et al., 2021; Pressler et al., 2019).

Fire activity in Sweden has varied considerably over the last 700 years, due to variation in climate and human activities (Drobyshev et al., 2015). Over the last century, fire has been rare in the landscape, because of efficient fire suppression (Granström, 2001), resulting in multiple changes in ecosystem structure and functioning (Nilsson & Wardle, 2005). Higher risks of fire and extended fire seasons are projected for Sweden in the future as a consequence of climate warming (Sjöström & Granström, 2020). In the unusually warm summers of 2014 and 2018, Sweden experienced two major wildfire seasons, which were anomalies relative to the preceding century (Drobyshev et al., 2021). The fire events in those years included not only large areas of ground fires but also stand‐replacing crown fires, which occurred mainly in young and intermediate‐aged *P. sylvestris* managed forests (Gustafsson et al., 2019). Although two thirds of the boreal forest are managed (Gauthier et al., 2015), most of our current understanding of how fire impacts boreal forests in northern Europe is derived from natural, uneven‐aged forest ecosystems. The introduction of large‐scale even‐aged rotational forestry (Östlund et al., 1997) has caused fundamental changes in forest ecosystem structure and functioning (Savilaakso et al., 2021). Thus, quantitative data on how managed forests sustain and recover from recent fire disturbance is central for predicting ecosystem resilience (Senf et al., 2019).

**FIGURE 1:**
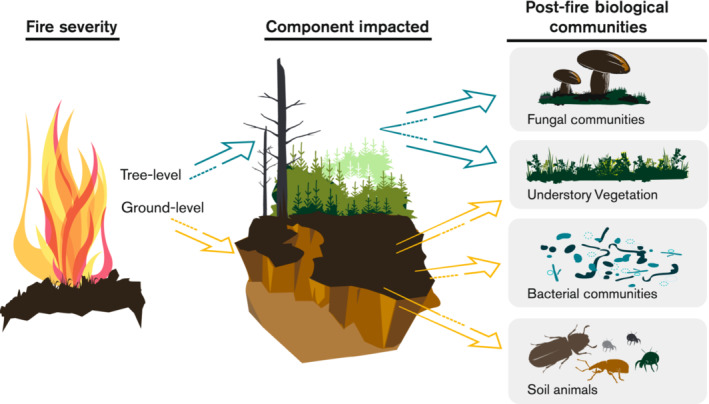
Conceptual diagram showing how we hypothesize that two separate fire severity gradients will affect biological communities: one aboveground gradient that is mainly attributed to damage of the trees and one gradient attributed to damage on the forest floor.

Here, we investigated ecosystem responses to a major wildfire known as the “Västmanland burn” that burned circa 13,000 ha of primarily even‐aged managed forest stands in Sweden in 2014. We studied forest stands that had been exposed to varying levels of fire severity, and nearby stands that escaped the fire. In 2016, we collected data on plant, soil microbial, and soil fauna communities in each unburned and burned stand to investigate responses to fire severity (sensu Keeley, 2009) while distinguishing between tree‐related (i.e., tree mortality) and soil‐related (i.e., consumption of soil organic matter) severity aspects. We expected that tree‐related and soil‐related fire severity would affect survival and recovery of communities of understory plants and soil biota differently, because of contrasting effects on the resources and habitats for these communities (Neary et al., 1999; Schimmel & Granström, 1996; Turner et al., 1999) (see Figure 1).

We tested three hypotheses. First, we hypothesized that the severity of the fire influences how the understory vegetation develops after the fire. Specifically, we expected fast‐growing plants to respond positively to extensive tree mortality because of competitive release from light and soil nutrient resources (Richter et al., 2019; Suding & Goldberg, 2001), while the response of slow‐growing plants would be determined by the depth of the remaining soil organic layer, as well as the ability of species to resprout from surviving rhizomes or to recolonize disturbed soils by seeds (Greene et al., 2004; Schimmel & Granström, 1996). Second, we hypothesized that soil fungi and bacteria would show contrasting responses to tree‐related and soil‐related fire severity. Specifically, we expected that soil fungi, being driven largely by inputs of recently fixed C from dominant plants, would depend on the survival of overstory trees (as presented in Pérez‐Izquierdo et al., 2021). In contrast, we expected that soil bacteria, which are more versatile in their C usage and less dependent on interactions with trees, would be more responsive to changes in soil C stocks and pH (Mataix‐Solera et al., 2009; Sun et al., 2016; Xiang et al., 2014). Third, we hypothesized that soil animals (microarthropods and nematodes) would be more affected by soil‐related aspects of fire severity than by tree‐related fire severity, because they mainly respond to changes in soil C stocks and habitat loss resulting from consumption of the organic horizon (Malmström, 2010; Rousseau et al., 2018). By addressing these hypotheses in combination, we provide insights about the initial responses and resilience of aboveground and belowground communities to variation in different aspects of fire severity in managed boreal forests, which is critical to advance understanding of the consequences of increased frequency of severe fires expected to result from climate warming.

## 2 MATERIALS AND METHODS

### 2.1 Description of the burn

During a severe summer drought in July 2014, a large wildfire affected 13,100 hectares of land in east‐central Sweden (59°53′44″ N, 16°1431′27″ E), making this fire the largest wildfire during the past 40 years in Sweden (Bohlin et al., 2017; Nilsson et al., 2014). Within the fire boundary, nearly 25% of the area experienced high‐intensity crown fires that killed the majority of the trees, whereas the remaining area was subjected to less intense crown fires or creeping ground fires that resulted in relatively less visible damage to the trees (see Gustafsson et al., 2019 for details). Thus, the fire created a mosaic of forest stands that were differentially affected by both crown fire and ground fire (Figure 1).

### 2.2 Stand selection

**FIGURE 2:**
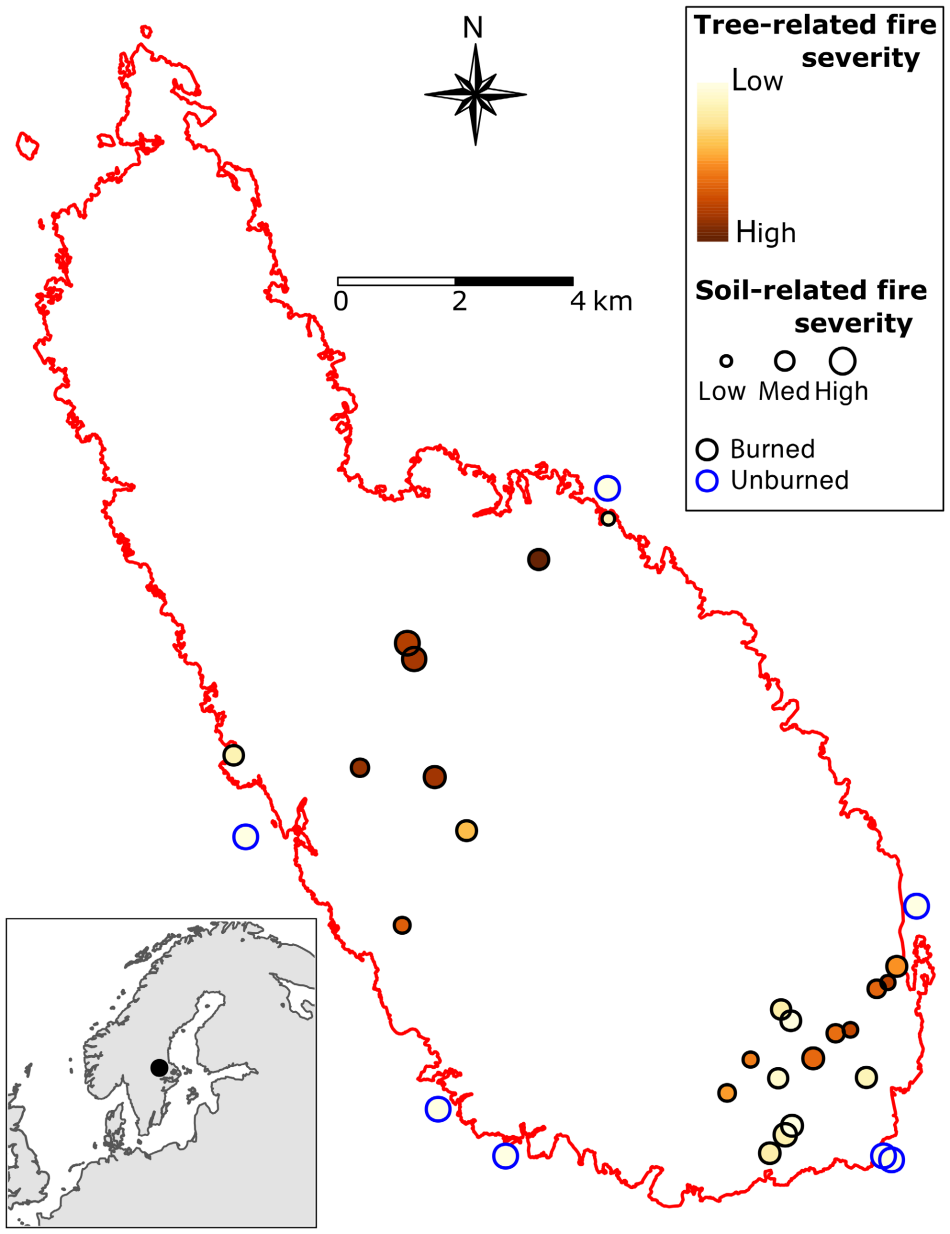
Map of the Västmanland burn that occurred in Sweden in 2014, and of the physical location of each of the 32 individual forest stands sampled in this study. Red contour line represents the fire boundary. Black outer line dots represent 25 individual burned stands and blue outer line dots represent the seven unburned reference stands. Circles corresponding to burned plots are colored according to tree‐related fire severity (from yellow [low] to brown [high]) and sized according to soil‐related fire severity. GPS coordinates for each stand are given in Table S1.

In November 2015, we selected 50 even‐aged (47 ± 6 year old) homogenous *Pinus sylvestris* stands within the fire boundary, with a mean area of 0.5 hectares, which before the fire had been managed with the purpose of biomass production. The stands were selected based on information from the land‐owner's data base about tree species composition, stand age, and site productivity (site index). All stands had to exhibit impacts from fire to the canopy and/or the soil surface or exhibit evidence of fire effects visible in satellite photos, as well as being easily accessible from forest roads. After a field visit, we finally selected 25 stands (out of the preselected 50 stands) with homogenous stand characteristic. All the selected stands were on level ground, had the same underlying geological parent material, had a site productivity index of T22–24 (i.e., a predicted height of trees of 22–24 m at an age of 100 years) and were dominated by mid‐aged *P. sylvestris* trees with some scattered *Picea abies*, *Betula pendula*, and *B. pubescens* trees. Prior to the fire, all stands had undergone thinning operations, and at the time of the fire stand densities were lower than 1000 stems per hectare. Stands were selected to represent a large gradient in fire severity, which included stands with trees that were severely burned (all trees were dead), moderately burned stands, and stands with trees that were only lightly affected by the fire (Figure S1). We also required that all selected stands had to exhibit at least some significant degree of ground fire as judged by visual inspection of burn effects on shrubs and soil organic matter layer. Within 500 m of the fire perimeter, we also selected seven unburned reference stands with similar underlying soil parent material, prefire stand age, management history, and elevation (i.e., 100 m a.s.l.) to the other stands (Figure 2) (Hägglund & Lundmark, 1999). All 32 stands (burned and unburned) were located within a 26 × 23 km area and were interspersed as much as possible over the whole area to reduce the effect of spatial autocorrelation (Figure 2).

### 2.3 Data collection

In April 2016, a circular plot (radius = 10 m) was established and permanently marked in the center of each of the burned and unburned stands for collection of postfire variables to describe the impact of fire (see below).

#### 2.3.1 Tree layer characteristics and litter and charcoal deposition

Within each circular plot, we recorded the number of live and dead trees (including snags and logs created by the fire), and measured tree diameter at breast height (DBH; 1.3 m) to calculate postfire tree density and basal area (m^2^ ha^−1^) for live and dead *P. sylvestris*, *P. abies*, *B. pendula*, and *B. pubescens* trees. Among these, the dominant trees *P. sylvestris* and *P. abies* are non‐resprouting species, while *B. pendula* and *B. pubescens* are capable of resprouting from the stem base. Tree diameter was further used to calculate tree biomass per area (kg ha^−1^) for each species using specific allometric functions (Marklund, 1988). These functions were used to estimate the biomass of stems, twigs, and branches for each tree species in the plot, as well as stump and coarse root biomass for *P. sylvestris* and *P. abies*. Carbon stocks of trees were calculated by multiplying total biomass by 50.3% C for *P. sylvestris*, 49.2% C for *P. abies*, and 49.4% C for *Betula* spp. (Lachowicz et al., 2018; Sandström et al., 2007). Viability (and mortality) of all standing trees was determined through visual inspection for the presence of green foliage (including resprouts of *Betula* spp.), and the presence of bark scorches were noted for each tree. In each circular plot, the proportion of canopy openings (i.e., light transmission) were determined from eight hemispherical photographs averaged per plot. Each circular plot was then divided in four subplot sectors directed to the four main compass directions. Tree height and mean stem soot height (indicative of flame height and fire intensity; Keeley, 2009) was measured for five standing trees in each sector. The level of tree crown scorches (loss of smaller branches and needles) was also estimated for each tree as the % (to the nearest 10%) of the total tree crown volume scorched. We also collected all the charcoal on the soil surface and brown needle litter on the ground (deposited after the fire) from four circular subplots (16 cm diameter) in each circular plot. Charcoal and needles were oven dried at 80°C for 48 h and weighed. These needle samples were not used to calculate organic stocks, but used as an additional indication of fire severity, with a larger amount of persisting needles indicating lower fire severity (Keeley, 2009).

#### 2.3.2 Soil properties

In September 2016, in each of the 32 stands, 25 soil cores (3 cm in diameter) were collected at 5 m intervals in a 20 × 20 m grid that overlapped the 10‐m radius circular plot. The samples were collected to the full depth of the organic soil horizon and a further 5 cm into the upper mineral soil. To obtain a quantitative soil sample to represent each stand (g per m^2^), we strictly sampled at each of the 25 grid points irrespective of whether one or both of the soil layers sometimes were missing by burned; note also that the organic horizon often covered bedrock or boulders and no mineral layer could be sampled. The cores were split into the organic horizon (including litter components) and underlying mineral horizon. Upon collection, soil samples from the same horizon were pooled and mixed, resulting in two composite samples per stand and a total of 64 soil samples. Samples were kept at −20°C and aliquots (~50 mL) were freeze‐dried and ball‐milled for subsequent analyses. Subsamples were analyzed for total carbon (C) and nitrogen (N) concentrations using an isotope ratio mass spectrometer (DeltaV, Thermo Fisher Scientific, Bremen, Germany) coupled to an Elemental analyzer (Flash EA 2000, Thermo Fisher Scientific, Bremen, Germany). The mass (dry weight) of the organic horizon per area was determined based on the added area of the 25 cores, and soil C and N stocks in the organic horizon were derived by multiplying with C and N concentrations. The depth of total and charred organic horizon was recorded in each sampling hole to serve as an indicator of organic layer consumption by the fire (Keeley, 2009). Bulk density of the organic horizon of each stand was determined by dividing the mass of collected organic horizon by the volume of the 25 cores. In addition, soil pH was measured in each sample in a 1:3 (weight: volume) soil‐to‐deionized water slurry using a 744 pH meter (Metrohm, Herisau, Switzerland).

#### 2.3.3 Root biomass

Data on fine root biomass (g m^−2^) of *P. sylvestris* and *Betula* spp. were obtained from Pérez‐Izquierdo et al. (2019), who conducted quantitative PCRs with tree species‐specific primers based on DNA extracts from the same soil samples as used in this study. Root DNA of *P. sylvestris* was found to be a robust proxy for live tree root biomass, and therefore included in this study as another measure of fire impact.

### 2.4 Responses of biological communities to fire

#### 2.4.1 Understory vegetation

Understory vegetation cover was assessed in each stand on 8–20 August 2016 using eight 0.5 × 0.5 m randomly located subplots within the 10‐m radius circular plot. In each of these subplots, 25 squares of 10 × 10 cm were designated, and within each square, the presence versus absence of each understory plant species (including newly established tree seedlings) was recorded. For each stand, the occurrence of each plant species within that stand was expressed as the average frequency (i.e., the proportion of the 8 × 25 squares in which each species was present).

#### 2.4.2 Microbial biomass and community structure

In the organic and mineral horizons, ergosterol, previously quantified in the same samples by Pérez‐Izquierdo et al. (2021), was used as a proxy of fungal biomass. Further, microbial phospholipid fatty acids (PLFAs) were measured for each of the 64 soil samples using the methods described by Bligh and Dyer (1959) and Mcintosh et al. (2012) and used as a proxy of both fungal and bacterial biomass. A total of 28 PLFA markers were detected that could be grouped in subsets of microbial PLFAs representing different functional groups. Total bacteria were represented by i‐15:0, a‐15:0, 15:0, i‐16:0, 16:1ɷ9, 16:1ɷ7, 16:0, i‐17:0, cy‐17:0, a‐17:0, 18:1ɷ7, and cy‐19:0 bacterial PLFAs (Frostegård & Bååth, 1996). Branched fatty acids i‐15:0, a‐15:0, i‐16:0, i‐17:0, and a‐17:0 were used as a measure of Gram‐positive bacteria while cy‐17:0, cy‐19:0, and 18:1ɷ7 represented Gram‐negative bacteria. Actinomycete contribution to biomass was estimated by the branched fatty acids 10me16:0, 10me17:0, and 10me18:0, whereas 18:2ɷ6 was used to estimate the contribution of fungi. Further, for comparisons with other biota, data on fungal community composition was obtained from Pérez‐Izquierdo et al. (2021), who conducted sequencing of ITS2 markers, amplified from DNA extracts from the same soil samples as used in this study.

#### 2.4.3 Soil animals

During 20–22 September 2016, soil microarthropods, that is, springtails (Collembola) and mites (Acari: Oribatida) were collected. We focused on Oribatida because they are the most abundant mite group in soils, and for both groups (i.e., Oribatida and Collembola), good taxonomic knowledge enables resolution to at least the genus level, and there is knowledge about their dispersal and succession when (re)colonizing habitats. Microarthropods were collected within four 10 × 10 cm squares in each circular plot. For this, a metal frame was pushed 10 cm into the ground and all the soil (including the litter layer) was collected. At the same time, four soil cores (diameter of 2.3 cm) were taken down to 10 cm to sample nematodes. The four samples for each faunal group were kept separate and all samples were stored cold (approx. +4°), brought to the laboratory and, within a week extracted for 4 days using modified Tullgren funnels (microarthropods) or for 24 h using modified Baermann funnels (nematodes). The extracted animals were determined to species (Collembola, Oribatida) or genera and feeding groups (nematodes). Because of time constraints, only a subset of sampling sites was analyzed for soil animals, viz. 4 unburned stands and 14 burned stands. These were chosen to be evenly distributed along the identified fire severity gradient (Figure 2, Table S1).

### 2.5 Data and statistical analyses

To assess whether the unburned forests were suitable as a proxy for stand characteristics of the burned stands before the fire, the prefire stand structures of the burned forest stands were reconstructed from the tree layer data (i.e., the total number of stems, tree height, basal area, and tree biomass of both live and dead trees present in each stand). General linear models (GLMs) were used to test for a systematic divergence between the 25 burned stands and the seven unburned (reference) stands. These models showed that, for most of the variables, the unburned stands were similar in structure to the burned stands (except that the unburned stands had 23% lower tree density and 12% larger diameter at breast height, as well as a slightly larger biomass proportion of *P. abies*) (Table S2). Moreover, to check for spatial autocorrelation of pre‐fire stand characteristics (i.e., stand age, tree basal area, tree height, total number of trees, diameter, and tree biomass) among burned and unburned stands, we performed a Mantel test of correlations between geographical distance (latitude and longitude) and similarities in stand characteristics. No significant spatial correlations were detected when all stands were considered (*r* = 0.094, *p* = 0.10).

To explore collinearity among all possible pairwise combinations of tree and soil variables, Pearson correlation analyses with hierarchical clustering were performed with the 25 burned stands serving as independent data points (Figure S2). Included variables were the proportion of dead trees (i.e., tree mortality) and stems scorched within each stand, stem soot height, proportion of crown scorched, the amount of deposited needle litter and charred litter on the ground as well as measurements of the organic soil horizon, that is, pH, C and N stocks, C:N ratio, and depth of the organic soil horizon. In addition, we included the depth of the charred organic horizon and root biomass of pine and birch. All variables were tested for normality and log or square root transformed when needed.

**FIGURE 3:**
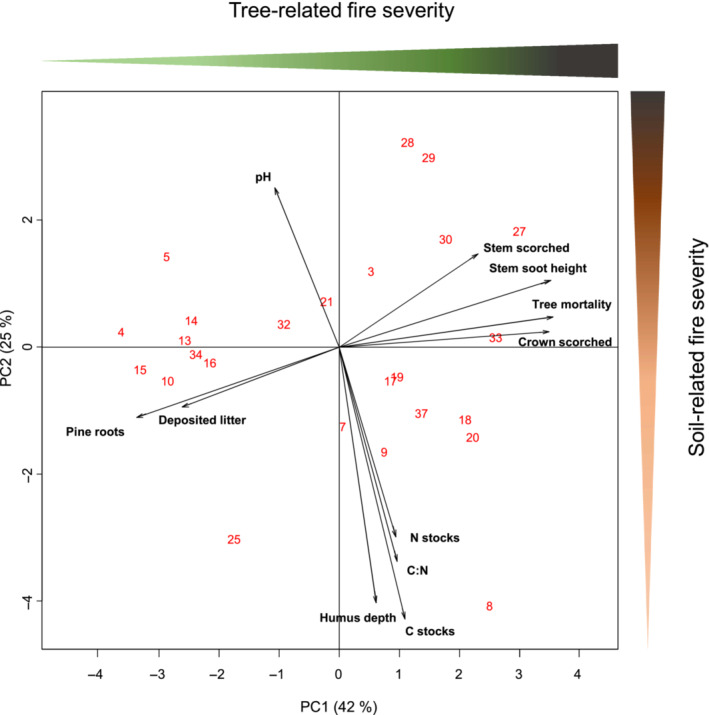
Principal component analysis (PCA) of aboveground and belowground variables that are indicative of tree‐related fire severity and soil‐related fire severity, respectively. The first two axes explaining the largest proportions of total variation (in parenthesis) are shown. Numbers represent forest stand IDs (Table S1). Mean values of each variable can be found in Table S4.

To visualize how the various indicators of fire severity were related to each other, a principal component analysis (PCA) was performed based on the 25 burned stands, which included the following variables: pH, proportion of scorched stems, stem soot height, proportion of scorched crown, soil organic C and N stocks, C:N, depth of the organic soil horizon, pine root biomass, deposited needle litter, and tree mortality. To determine the number of components or factors to retain from the PCA, we performed a Horn's parallel analysis implemented in the paran R package. We observed a clear dichotomy in fire impacts, where indicators of tree‐related impact were aligned along the primary ordination axis (PC1; hereafter referred to as tree‐related fire severity) and indicators of soil‐related effects (mainly soil abiotic properties) were aligned along the secondary ordination axis (PC2); hereafter referred as to soil‐related fire severity; Figure 3, Figure S3. All of the collected tree and soil data are typical metrics of fire severity (Keeley, 2009). The third axis of the PCA (Table S3, Figure S3) could be interpreted as a proxy of soil fertility. Thus, two separate fire severity gradients were evaluated: one mainly attributed to the fire effects on the trees, and one to the effects of fire on the ground. The fertility gradient, which related to intrinsic characteristics of the plots, was not addressed in subsequent analyses because of lack of statistical significance as predictor of biotic variation.

Differences between the seven unburned stands and the 25 burned stands were assessed using GLMs for stand descriptive variables, measures of the organic and mineral horizons of the soil, Shannon's diversity index (H), richness (S) and frequency of vascular and nonvascular plant species, microbial biomass (measured using both PLFA and ergosterol in organic and mineral soil), and richness and abundances of soil faunal groups. The effect of fire (unburned vs burned stands) on the community composition of understory vegetation and of soil fungi were analyzed by PERMANOVA of Bray–Curtis dissimilarity matrices using the *adonis* function of the Vegan R package.

Effects of tree‐related and soil‐related fire severity on compositional community responses of each organismal group were evaluated using GLMs, in which tree‐related fire severity and soil‐related fire severity (i.e., the scores on the first two axes of the PCA) were included as explanatory variables. The response variables included in separate models were Shannon diversity index, richness and frequency of vascular and nonvascular plant species, microbial biomass (ergosterol and different groups of PLFAs in the organic and mineral soil), and richness and abundances of animal groups. The relative importance of tree‐related and soil‐related fire severity effects was tested by the averaging over orderings method (lmg) implemented in the Relaimpo R package and bootstrap confidence intervals were calculated. Further, effects of tree‐related and soil‐related fire severity on community composition were visualized through nonmetric multidimensional scaling (NMDS) biplots performed on Bray–Curtis dissimilarity matrices of understory vegetation, soil fungal and nematode communities and on Euclidean dissimilarity matrices of bacteria PLFAs. Tree‐related fire severity and soil‐related fire severity ordinates were plotted and correlations with communities were statistically evaluated with the *envfit* and *metaMDS* functions in the Vegan R package. All analyses were performed in R version 4.1.1 (R Core Team, 2021).

## 3 RESULTS

### 3.1 Forest stand characteristics in response to fire

All trees present in each plot before the fire were identifiable after the fire, either as live standing trees, or as snags or logs. We did not observe any traces of stumps or roots that would have indicated complete combustion of large trees or any dead wood that could have been present before the fire. Compared to prefire stem density levels (i.e., the sum of the number of live trees, snags and logs within each plot), postfire survival was 32% for *P. sylvestris*, 1% for *P. abies*, and 45% for *Betula* spp. across all burned stands (Table S4). The majority of trees killed by the fire remained as postfire snags after 2 years, while <13% of the trees were observed as postfire logs (Table S4). About 40% (i.e., 22.9 ± 5.8 Mg C ha^−1^) of the total prefire tree biomass remained in living *P. sylvestris* postfire. As presented in Pérez‐Izquierdo et al. (2019), the fire also caused a 97% loss of *P. sylvestris* fine root biomass across all burned stands, but there was no significant effect on the abundance of live *Betula* spp. roots 2 years after the fire (Table S4).

On average, the fire generated a stem soot height of 4.0 ± 0.6 m and scorched 72 ± 7% of the total crown volume (71 ± 7% pine, 100 ± 0% spruce and birch) (Table S4). Light transmission through the tree canopy varied from 73% to 94% in the burned stands and was on average 66% in unburned stands (Table S4). The mass of postfire deposited litter was 631 ± 66 g m^−2^ and consisted mainly of *P. sylvestris* needles affected by heat or postfire tree mortality. The average amount of charred litter (≤4 mm) deposited on the forest floor was 118 ± 22 g m^−2^ (Table S4).

Soil pH in the organic horizon was increased by almost one unit after the fire, and the bulk density was three times higher (Table 1). The depth of the organic soil horizon varied from 10 to 42 mm in the burned stands and was on average 94 mm in unburned stands. The N and C stocks of the organic horizon were 29% and 46% lower, respectively, in burned than in unburned stands, and the C:N ratio was 25% lower in burned stands (Table 1). Effects of fire on mineral soil properties were negligible (Table 1).

**TABLE 1:** Characteristics of the organic and mineral soil layer (Mean ± SE) in burned and unburned forest stands.

	Burned forests (n = 25)	Unburned forests (*n* = 7)	*t* (*p*‐value)
**Organic soil**			
pH^a^	4.7 ± 0.1	4.0 ± 0.1	**6.63 (<0.001)**
Depth (mm)^a^	25.2 ± 1.7	94.0 ± 5.5	**−16.10 (<0.001)**
Bulk density (g m^3^)	0.29 ± 0.04	0.09 ± 0.01	**2.54 (0.017)**
Total C (kg m^−2^)^a^	1.878 ± 0.100	3.508 ± 0.273	**−6.67 (<0.001)**
Total N (kg m^−2^)^a^	0.065 ± 0.003	0.091 ± 0.005	**−4.70 (<0.001)**
C:N^a^	28.8 ± 4.2	38.3 ± 3.5	**−5.55 (<0.001)**
Depth of charred organic horizon (mm)	12.8 ± 0.6	0 ± 0	
**Mineral soil**			
Total C (kg m^−2^)^a^	0.782 ± 0.074	0.556 ± 0.100	1.44 (0.160)
Total N (kg m^−2^)^a^	0.023 ± 0.002	0.017 ± 0.003	1.45 (0.159)
C:N^a^	33.9 ± 0.9	32.7 ± 2.1	0.60 (0.553)

*Note*: Values of *t* and *p* are derived from General Linear Models testing for differences between burned and unburned forest stands. Significant differences (*p* < 0.05) are highlighted in bold.

^a^ Data from Pérez‐Izquierdo et al., 2021.

### 3.2 Responses of biological communities to fire severity

#### 3.2.1 Understory vegetation

**FIGURE 4:**
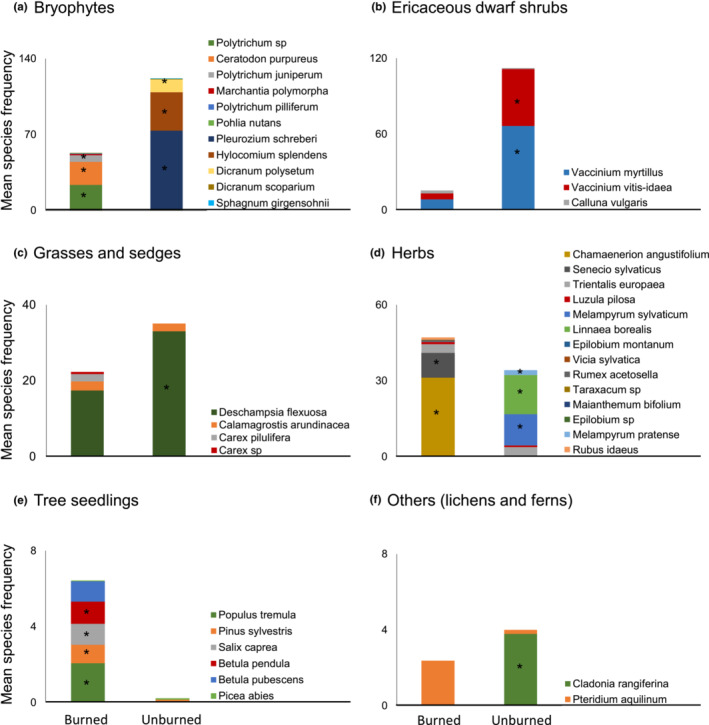
Mean frequency of species of (a) bryophytes, (b) ericaceous dwarf shrubs, (c) grasses and sedges, (d) herbs, (e) newly established tree seedlings, and (f) others (lichen and ferns) that are present in more than one stand in the understory of the burned and unburned forest stands. Asterisks indicate significant differences for individual species between burned and unburned forests (results of the statistical tests in Table S5).

Two years after the fire, the composition of understory vegetation of unburned and burned stands differed greatly (PERMANOVA: *R* ^2^ = 0.56; Pseudo‐*F* = 37.8; *p* < 0.001). Understory communities in burned stands had a significantly higher Shannon's diversity index than those in unburned stands, while the total plant species richness did not differ (Table S5). Plant communities in unburned stands were dominated by the mosses *Hylocomium splendens* and *Pleurozium schreberi*, the ericaceous dwarf shrubs *Vaccinium myrtillus* and *V. vitis‐idaea*, the grass *Deschampsia flexuosa* and the vascular plant *Linnaea borealis* (Figure 4; Table S5). In contrast, the vegetation of burned forests was composed mainly of typical fire‐ or disturbance‐favored species, such as the mosses *Polytrichum* spp. and *Ceratodon purpureus* and the herb *Chamaenerion angustifolium*. In the burned forest, there were also significantly more regenerating tree seedlings, especially of *P. sylvestris* and the deciduous *B. pendula*, *Populus tremula*, and *Salix caprea* (Table S5).

**FIGURE 5:**
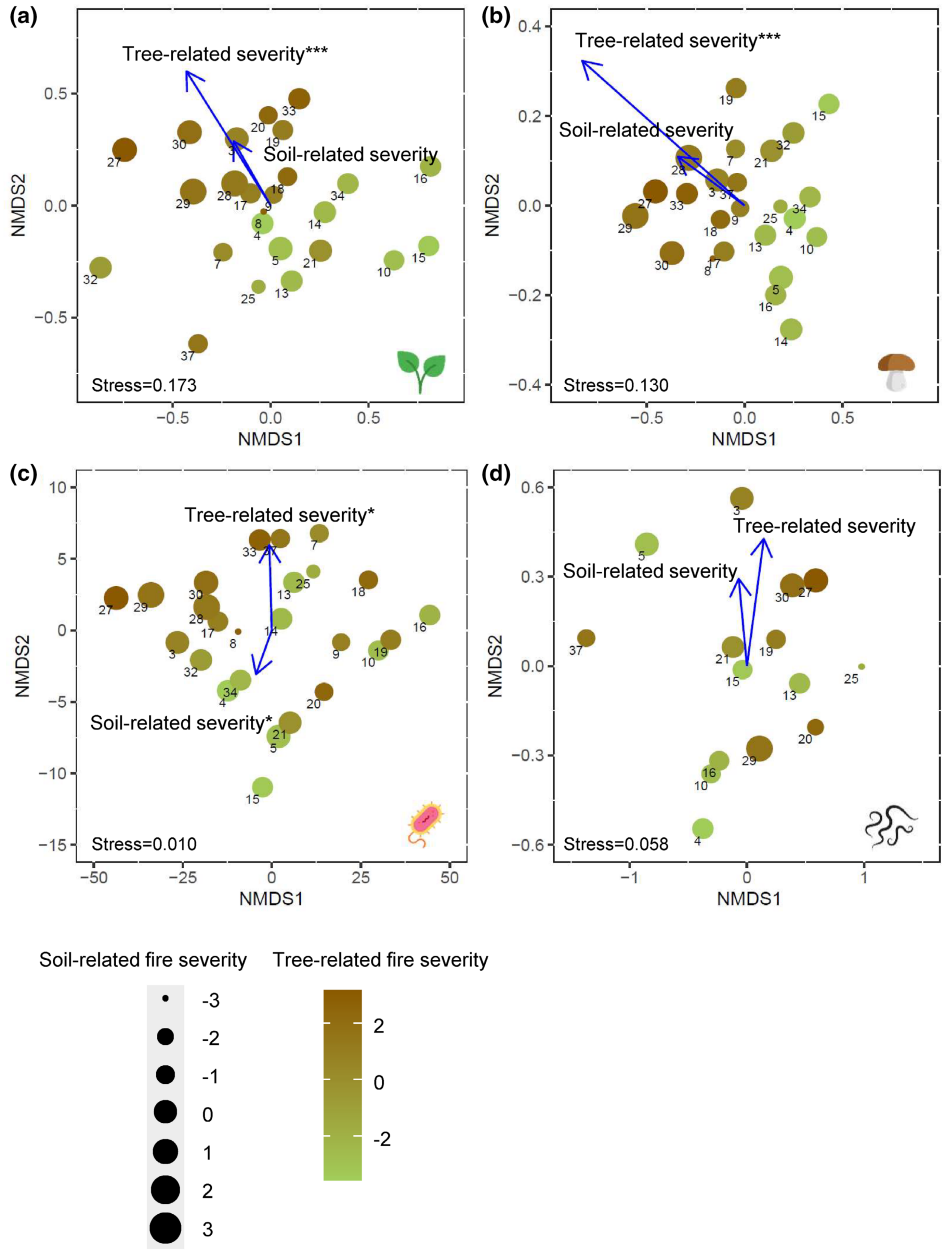
Ordination analysis of response variables displayed by NMDS for community composition of (a) understory vegetation, (b) soil fungi, (c) soil bacterial PLFAs, and (d) soil Nematoda. Vectors indicate the direction and magnitude of correlations of community assemblage with tree‐related fire severity and soil‐related fire severity indexes. Asterisks denote level of significant correlations (*p < 0.05, **p < 0.01, and ***p < 0.001). Stress values shown at the bottom of each graph correspond to two dimensions (*k* = 2). Numbers represent forest stand IDs (Table S1).

The overall composition of understory vegetation correlated significantly with tree‐related fire severity but not with soil‐related fire severity (Figure 5a, Figure S4). The total species richness and the Shannon's diversity index for the understory vegetation did not respond to fire severity (Table 2). In particular, high tree‐related fire severity hampered natural regeneration of *P. sylvestris* and *B. pubescens* and reduced the occurrence of the ericaceous dwarf‐shrub *V. vitis‐idaea* and the grass *D. flexuosa*, while the two mosses *C. purpureus* and *Polytrichum juniperinum* were positively affected (Table 2).

**TABLE 2:** Results of General Linear Models (GLMs) (*t*‐values with *p*‐values in brackets) testing for the effects of tree‐related fire severity and soil‐related fire severity, and the relative importance of these two predictors tested using the “averaging over orderings” method (lmg) (mean estimates with 95% bootstrapping confidence intervals in brackets), on plant community variables in the understory of the burned forest stands.

	GLM		lmg	
	Severity		Severity	
	Tree‐related	Soil‐related	Tree‐related	Soil‐related
Shannon's index of diversity (H)	−1.36 (0.187)	−1.80 (0.087)	0.069 [0.001, 0.341]	0.119 [0.002, 0.399]
Species richness (S)	−1.05 (0.304)	−0.94 (0.356)	0.046 [0.000, 0.346]	0.037 [0.001, 0.260]
Species frequency^a^:				
Herbs				
*Chamaenerion angustifolium*	0.86 (0.399)	0.70 (0.493)	0.032 [0.000, 0.321]	0.021 [0.000, 0.128]
*Luzula pilosa*	0.20 (0.845)	0.43 (0.669)	0.002 [0.000, 0.286]	0.009 [0.000, 0.198]
*Rubus idaeus*	0.57 (0.575)	0.88 (0.390)	0.014 [0.000, 0.253]	0.033 [0.000, 0.275]
*Senecio sylvaticus*	−0.12 (0.906)	**2.91 (0.008)**	0.001 [0.000, 0.203]	0.278 [0.107, 0.534]
*Trientalis europaea*	0.18 (0.862)	−0.64 (0.529)	0.001 [0.000, 0.303]	0.018 [0.000, 0.168]
b Ericaceous dwarf shrubs				
*Calluna vulgaris*	−1.28 (0.213)	−0.25 (0.809)	0.069 [0.001, 0.434]	0.003 [0.000, 0.155]
*Vaccinium myrtillus*	−0.21 (0.834)	**−2.52 (0.019)**	0.002 [0.001, 0.195]	0.223 [0.037, 0.508]
*Vaccinium vitis‐idaea*	**−2.49 (0.021)**	**−2.90 (0.008)**	0.169 [0.009, 0.436]	0.230 [0.051, 0.481]
c Grasses and sedges				
*Calamagrostis arundinacea*	−1.52 (0.142)	−0.54 (0.597)	0.094 [0.001, 0.416]	0.012 [0.000, 0.138]
*Carex pilulifera*	0.47 (0.646)	1.10 (0.282)	0.009 [0.000, 0.193]	0.052 [0.001, 0.300]
*Deschampsia flexuosa*	**−4.75 (<0.001)**	−0.55 (0.586)	0.503 [0.281, 0.694]	0.007 [0.001, 0.106]
d Tree seedlings^b^				
*Betula pendula*	0.06 (0.953)	−1.68 (0.108)	0.000 [0.000, 0.153]	0.113 [0.003, 0.429]
*Betula pubescens*	**−2.16 (0.042)**	−0.27 (0.787)	0.175 [0.002, 0.575]	0.003 [0.000, 0.162]
*Pinus sylvestris*	**−2.75 (0.012)**	−1.56 (0.134)	0.237 [0.013, 0.596]	0.076 [0.001, 0.302]
*Populus tremula*	0.78 (0.446)	−0.38 (0.707)	0.027 [0.000, 0.227]	0.006 [0.000, 0.238]
*Salix caprea*	−0.12 (0.905)	0.38 (0.706)	0.001 [0.000, 0.231]	0.007 [0.000, 0.170]
e Ferns				
*Pteridium aquilinum*	1.73 (0.097)	−1.01 (0.326)	0.001 [0.000, 0.231]	0.007 [0.000, 0.170]
f Bryophytes				
*Ceratodon purpureus*	**2.50 (0.020)**	1.93 (0.067)	0.196 [0.012, 0.468]	0.116 [0.005, 0.397]
*Marchantia polymorpha*	1.61 (0.123)	−1.44 (0.164)	0.098 [0.002, 0.349]	0.078 [0.004, 0.319]
*Pohlia nutans*	−0.31 (0.757)	0.11 (0.913)	0.005 [0.000, 0.199]	0.001 [0.000, 0.153]
*Polytrichum juniperinum*	**2.45 (0.023)**	**2.98 (0.007)**	0.163 [0.006, 0.391]	0.241 [0.022, 0.527]
*Polytrichum piliferum*	−0.90 (0.380)	−0.02 (0.987)	0.035 [0.000, 0.298]	0.000 [0.000, 0.154]
*Polytrichum* sp.	0.41 (0.689)	−0.28 (0.785)	0.007 [0.000, 0.231]	0.003 [0.000, 0.151]

*Note*: Significant values (*p* < 0.05) are highlighted in bold.

^a^ Average of eight quadrants per 10 m radius plot.

^b^ Newly established tree seedling from seeds.

#### 3.2.2 Microbial biomass and community structure

In the organic horizon, soil fungal community composition (from Pérez‐Izquierdo et al., 2021) differed between burned and unburned stands (PERMANOVA: *R* ^2^ = 0.28; Pseudo‐*F* = 11.5; *p* < 0.001). The fungal biomass measured by ergosterol or PLFA was about 60% lower in the burned than in the unburned stands (Table 3). Concentrations of PLFAs from bacteria were also 56% lower in the organic horizon of burned stands than unburned stands (Table 3). In the mineral soil, the differences were smaller, but also here, burned stands had lower fungal and bacterial PLFA concentrations compared to unburned stands (Table 3). The fungal to bacterial ratio did not differ between burned and unburned stands neither in the organic horizon nor in the mineral soil (Table 3).

**TABLE 3:** Concentrations of microbial PFLAs and ergosterol (Means ± SE) from organic and mineral soils in the burned and unburned forest stands.

	Burned forests (*n* = 25)	Unburned forests (*n* = 7)	*t* (*p*‐value)
**Organic soil**			
Ergosterol (μg per g soil dry weight^−1^)^a^	22.6 ± 1.6	54.8 ± 5.7	**−6.5 (<0.001)**
PLFAs (nmol per g soil dry weight^−1^)			
Fungi	111.3 ± 6.8	281 ± 18	**−7.1 (<0.001)**
Bacteria	181 ± 12	414 ± 15	**−8.0 (<0.001)**
Gram‐positive bacteria	63.5 ± 4.4	145.3 ± 4.2	**−5.8 (<0.001)**
Gram‐negative bacteria	77.7 ± 4.8	176.7 ± 9.6	**−6.4 (<0.001)**
Actinomycetes	35.4 ± 2.7	81.3 ± 4.9	**−5.4 (<0.001)**
Fungal to bacterial ratio	0.63 ± 0.02	0.68 ± 0.05	−1.0 (0.304)
**Mineral soil**			
Ergosterol (μg per g soil dry weight^−1^)^a^	1.7 ± 0.1	4.1 ± 0.4	**−6.5 (<0.001)**
PLFAs (nmol per g soil dry weight^−1^)			
Fungi	22.8 ± 1.8	34.3 ± 5.3	**−2.4 (0.023)**
Bacteria	74.1 ± 5.4	102 ± 10	**−2.5 (0.020)**
Gram‐positive bacteria	24.4 ± 1.8	33.7 ± 3.6	**−2.4 (0.023)**
Gram‐negative bacteria	36.6 ± 2.7	49.0 ± 5.6	**−2.1 (0.046)**
Actinomycetes	15.6 ± 1.1	23.2 ± 3.2	**−2.7 (0.010)**
Fungal to bacterial ratio	0.31 ± 0.01	0.34 ± 0.04	−1.0 (0.285)

*Note*: Values of *t* and *p* are derived from General Linear Models testing for differences between burned and unburned forest stands. Significant differences (*p* < 0.05) are highlighted in bold.

^a^ Data from Pérez‐Izquierdo et al., 2021.

Soil fungal community composition in the organic horizon correlated strongly with tree‐related fire severity but not with soil‐related fire severity (Figure 5b). Soil fungal biomass (measured both as ergosterol and fungal PLFAs) were negatively correlated with both tree‐related and soil‐related fire severity gradients (Table 4). The composition of bacterial PLFAs was related to both severity gradients (Figure 5c). Gram‐positive, Gram‐negative and actinomycete PLFAs were all negatively correlated with soil‐related fire severity while tree‐related fire severity marginally correlated with Gram‐positive and Gram‐negative PLFAs (Table 4). The ratio of fungi to bacteria of the organic horizon was unrelated to either of the severity gradients (Table 4). In the mineral soil, fungal biomass (as indicated by ergosterol) decreased significantly with increasing tree‐related fire severity (Table 4), while the fungal and bacterial PLFAs did not correlate with either of the severity gradients. However, the fungal to bacterial ratio decreased with increasing soil‐related fire severity (Table 4).

**TABLE 4:** Results of General Linear Models (GLMs) (*t*‐values with *p*‐values in brackets) testing for the effects of tree‐related fire severity and soil‐related fire severity, and the relative importance of these two predictors tested using the “averaging over orderings” method (lmg) (mean estimates with 95% bootstrapping confidence intervals in brackets), on concentrations of PLFAs and ergosterol content in the organic and mineral soil layers.

	GLM		lmg	
	Severity		Severity	
	Tree‐related	Soil‐related	Tree‐related	Soil‐related
**Organic soil**				
Ergosterol^a^	**−3.77 (0.001)**	**−2.61 (0.016)**	0.342 [0.079, 0.619]	0.143 [0.009, 0.477]
PLFAs^b^:				
Fungi	**−3.56 (0.002)**	**−3.30 (0.003)**	0.279 [0.070, 0.527]	0.238 [0.071, 0.522]
Bacteria	−1.90 (0.071)	**−3.30 (0.003)**	0.099 [0.002, 0.309]	0.298 [0.072, 0.605]
Gram‐positive bacteria	−1.91 (0.069)	**−3.52 (0.002)**	0.096 [0.002, 0.303]	0.325 [0.078, 0.637]
Gram‐negative bacteria	−2.05 (0.052)	**−2.05 (0.004)**	0.114 [0.002, 0.344]	0.288 [0.074, 0.563]
Actinomycetes	−1.35 (0.191)	**−3.67 (0.001)**	0.049 [0.001, 0.250]	0.361 [0.117, 0.651]
Fungi to bacteria ratio	−1.37 (0.184)	0.99 (0.330)	0.076 [0.001, 0.396]	0.040 [0.001, 0.380]
**Mineral soil**				
Ergosterol^a^	**−2.37 (0.027)**	−1.28 (0.213)	0.192 [0.009, 0.571]	0.056 [0.002, 0.250]
PLFAs^b^:				
Fungi	−0.00 (0.998)	−1.96 (0.063)	0.000 [0.000, 0.224]	0.149 [0.004, 0.414]
Bacteria	0.09 (0.929)	−0.82 (0.423)	0.000 [0.000, 0.255]	0.029 [0.000, 0.285]
Gram‐positive bacteria	0.22 (0.832)	−0.81 (0.427)	0.002 [0.000, 0.244]	0.029 [0.000, 0.239]
Gram‐negative bacteria	0.20 (0.842)	−0.63 (0.537)	0.002 [0.000, 0.239]	0.013 [0.000, 0.272]
Actinomycetes	0.41 (0.691)	−1.44 (0.164)	0.007 [0.000, 0.263]	0.086 [0.002, 0.333]
Fungi to bacteria ratio	−0.19 (0.848)	**−3.29 (0.003)**	0.001 [0.001, 0.218]	0.330 [0.005, 0.628]

*Note*: Significant values (*p* < 0.05) are highlighted in bold.

^a^ Data from Pérez‐Izquierdo et al., 2021 and expressed in μg of ergosterol per g of soil dry weight^−1^.

^b^ PLFAs are expressed in nmol per g of soil dry weight^−1^.

#### 3.2.3 Soil animals

In burned stands, the total abundances of Oribatida, Collembola, and nematodes were 63%, 49%, and 59% lower, respectively, than in unburned stands (Table 5). Among the nematodes, plant root hair feeders and fungal feeders were significantly lower in burned stands, while obligate plant feeders, bacterial feeders, predators, and omnivores did not differ between burned and unburned stands (Table 5). Species richness of Oribatida was 40% lower in burned than in unburned stands, while species richness of Collembola and genus richness of nematodes did not differ (Table 5).

**TABLE 5:** Characteristics of soil faunal communities (Mean ± SE) in the burned and unburned forest stands.

	Burned forests (*n* = 14)	Unburned forests (*n* = 4)	*t* (*p*‐value)*
**Abundance**			
Oribatida^a^	90.7∙10^3^ ± 13.1.10^3^	244.6∙10^3^ ± 51.4∙10^3^	**−4.34 (<0.001)**
Collembola^a^	35.9∙10^3^ ± 6.6∙10^3^	69.9∙10^3^ ± 18.2∙10^3^	**−2.17 (0.044)**
Nematoda^b^	95 ± 26	233 ± 50	**−2.45 (0.025)**
Nematoda (functional groups):			
Obligate plant feeders^b^	2.08 ± 1.45	0.06 ± 0.06	0.68 (0.503)
Root hair feeders^b^	16.7 ± 6.6	52.1 ± 11.1	**−2.51 (0.022)**
Fungal feeders^b^	14.1 ± 8.2	83.1 ± 35.6	**−2.95 (0.009)**
Bacterial feeders^b^	55.5 ± 12.2	89.8 ± 20.6	−1.32 (0.204)
Predators^b^	1.0 ± 0.5	0.42 ± 0.3	0.57 (0.576)
Omnivores^b^	5.5 ± 1.7	7.5 ± 1.9	−0.57 (0.578)
**Richness**			
Oribatida (species)^c^	24.5 ± 1.9	40.75 ± 2.8	**−4.14 (<0.001)**
Collembola (species)^c^	19.7 ± 1.1	23.5 ± 1.5	−1.69 (0.110)
Nematoda (genera)	21.3 ± 1.1	25.8 ± 1.5	−1.90 (0.075)

*Note*: Values of *t* and *p* are derived from General Linear Models testing for differences between burned and unburned forest stands. Significant differences (*p* < 0.05) are highlighted in bold.

^a^ Abundance is expressed as individuals per m^2^.

^b^ Abundance is expressed as individuals per g soil.

^c^ Richness is species number on 400 cm^2^ area.

Species richness and total abundance of Oribatida were negatively correlated with tree‐related fire severity, but not with soil‐related fire severity, while Collembola and nematodes did not respond significantly to either of the fire severity gradients (Table 6). Neither tree‐related nor soil‐related fire severity affected the structure of the nematode community (Figure 5d). None of the functional groups of nematodes responded to any of the fire severity gradients (Table 6).

**TABLE 6:** Results of General Linear Models (GLMs) (*t*‐values with *p*‐values in brackets) testing for the effects of tree‐related fire severity and soil‐related fire severity, and the relative importance of these two predictors tested using the “averaging over orderings” method (lmg) (mean estimates with 95% bootstrapping confidence intervals in brackets), on soil faunal communities.

	GLM		lmg	
	Severity		Severity	
	Tree‐related	Soil‐related	Tree‐related	Soil‐related
**Abundance**				
Oribatida^a^	**−2.35 (0.037)**	−0.65 (0.529)	0.322 [0.019, 0.638]	0.059 [0.001, 0.429]
Collembola^a^	−0.38 (0.709)	−0.03 (0.980)	0.013 [0.000, 0.367]	0.001 [0.000, 0.186]
Nematoda^b^	−1.09 (0.299)	0.52 (0.611)	0.081 [0.001, 0.745]	0.012 [0.001, 0.520]
Nematoda (functional groups):				
Obligate plant feeders^b^	−0.26 (0.799)	−0.30 (0.769)	0.008 [0.000, 0.459]	0.010 [0.000, 0.361]
Root hair feeders^b^	−0.06 (0.952)	1.14 (0.278)	0.003 [0.001, 0.359]	0.099 [0.002, 0.656]
Fungal feeders^b^	0.03 (0.981)	0.46 (0.656)	0.001 [0.000, 0.415]	0.018 [0.000, 0.524]
Bacterial feeders^b^	−1.41 (0.185)	0.10 (0.925)	0.144 [0.001, 0.744]	0.004 [0.001, 0.508]
Predators^b^	−0.03 (0.974)	0.23 (0.823)	0.020 [0.001, 0.388]	0.001 [0.001, 0.362]
Omnivores^b^	0.03 (0.976)	−0.17 (0.870)	0.000 [0.000, 0.373]	0.002 [0.000, 0.278]
**Richness**				
Oribatida (species)^c^	**−2.97 (0.012)**	−1.24 (0.237)	0.408 [0.098, 0.673]	0.122 [0.005, 0.458]
Collembola (species)^c^	−1.04 (0.321)	−0.46 (0.657)	0.093 [0.002, 0.591]	0.030 [0.001, 0.349]
Nematoda (genera)	0.98 (0.348)	−0.30 (0.769)	0.070 [0.002, 0.403]	0.004 [0.001, 0.549]

*Note*: Significant values (*p* < 0.05) are highlighted in bold.

^a^ Abundance is expressed as individuals m^2^.

^b^ Abundance is expressed as individuals g soil^1^.

^c^ Richness is species number on 400 cm^2^ area.

## 4 DISCUSSION

In this study, we found that fire changed the composition of the understory vegetation and increased species diversity relative to unburned forests, in a manner that matches postfire changes in other boreal forests of northern Europe (Kuuluvainen & Rouvinen, 2000; Nilsson & Wardle, 2005; Schimmel & Granström, 1997). As such, fire typically promoted seed regeneration of *P. sylvestris* and the broadleaved species *B. pendula*, *P. tremula*, and *S. caprea* as well as the herbs *C. angustifolium* and *Senecio sylvaticus*. Likewise, fire profoundly affected microbial community composition and reduced microbial biomass. Burned stands had 55%–60% less fungal and bacterial biomass compared to unburned stands, similar to what was reported by Holden et al. (2016) in boreal forests of Alaska. Similarly, all studied soil faunal groups were negatively affected by the fire, with total abundances reduced by >50% compared to unburned forests, in line with several other studies of soil fauna following burning (Broza & Izhaki, 1997; Kim & Jung, 2008; Malmström et al., 2009; Whitford et al., 2014; Wikars & Schimmel, 2001). This indicates that the overall responses of biological communities to fire disturbance in managed *P. sylvestris* forests, at least in the short‐term, are similar to those of postfire successions of natural forests. Our estimates of prefire stand characteristics indicate that the burned forest were very similar, but not identical, to the non‐burned areas that we used as reference, and the results should be interpreted with this in mind. Within this work, we also identified different aspects of fire severity to have contrasting effects on the survival and short‐term postfire recovery of various organism groups, which is relevant for predicting the consequences of a changing fire regimes of boreal forests. We here discuss these patterns in relation to our hypotheses.

In agreement with our first hypothesis, fire severity impacted the short‐term, postfire response of plant community composition. While we found fire to support regeneration of tree seedlings relative to unburnt forests, we did not find tree seedlings (or any vascular plants) to be favored by high tree‐related fire severity. Instead, regeneration of *P. sylvestris* and *Betula* spp. showed a negative response to tree mortality. *Pinus sylvestris* is adapted to low‐intense ground fires and pulses of natural regeneration typically occur in the vicinity of surviving trees shortly after fire (Kuuluvainen, 2016). Extensive mortality and crown scorches of overstory trees therefore likely reduced the number of available pine seeds on site, leading to slower recolonization dependent on distant seed sources (Sannikov et al., 2021). The reduced presence of ectomycorrhizal fungi observed in stands with high tree mortality could also have reduced the access of tree seedlings to established mycorrhizal mycelia (Ibáñez et al., 2021), which could disfavor recolonization by both *P. sylvestris* and *Betula* spp. seedlings (Nara, 2005). Seed predation by herbivores could also be a significant factor in explaining reduced tree regeneration after severe fires (Tasker et al., 2011; Zwolak et al., 2010). The grass *D. flexuosa* is known as a fire‐favored species, capable of resprouting from rhizomes buried in the soil. However, *D. flexuosa* was disfavored by high fire severity in our study, as were the dwarf shrubs *V. vitis‐idaea* and *V. myrtillus*. Resprouters have been suggested to be less heat and drought tolerant than other plant species (Vilagrosa et al., 2014; Villén‐Pérez et al., 2020) and their rhizomes can occur higher up in the organic soil in a manner that is then consumed or killed by heat in a severe fire (Schimmel & Granström, 1996). The only plant species that were favored by high tree‐related fire severity were the bryophytes *C. purpureus* and *P. juniperinum*, which are known to occupy habitats with low competition and high light regimes (Glime, 2017). The only vascular plant species that were clearly favored by high soil‐related fire severity was the annual species *S. sylvaticus*, which has been found to be positively related to increased soil pH after fire (Gustafsson et al., 2021). Overall, this supports our prediction that slow‐growing ericaceous shrubs and species that are capable of vegetative regeneration should recover quickly following low fire severity.

Plant diversity 2 years postfire was overall uncorrelated with fire severity. This finding is somewhat surprising, but consistent with a study of dry mixed conifer forests in California (Richter et al., 2019), which found that the relationship between postfire recovery of understory plant diversity and fire severity can vary considerably depending on the specific conditions of the fire. In our study, the absence of a clear relationship may be explained through none of the severity gradients having encompassed a wide enough range to provide different niches for colonizing plants (Richter et al., 2019) or by a strong influence of stochastic processes in early postfire successions as suggested by Gustafsson et al. (2021).

Our results only partly supported our second hypothesis that soil fungi and bacteria would show contrasting responses to tree mortality and combustion of the organic soil layer, as tree‐related fire severity had a major impact on both soil fungi and bacteria. Tree‐related fire severity reduced soil fungal biomass and changed fungal community composition. The large decrease in ergosterol associated with high fire severity indicates a significant loss of ectomycorrhizal fungal biomass resulting from tree mortality. Likewise, the changes in soil fungal community composition that we observed in response to tree deaths were mainly due to losses of fungal taxa that are dependent on C delivered by trees in the relatively short‐term, such as ectomycorrhizal and litter‐associated fungi (Pérez‐Izquierdo et al., 2021). Meanwhile, high soil‐related fire severity reduced the abundance of opportunistic molds, which, like bacteria, benefit from a continual supply of more easily degradable organic compounds from various sources (Lindahl et al., 2010; Tláskal et al., 2016). These compounds can be released, for example, from dead mycelium and tree roots, as well as from flushes of other labile soil resources released by low severity ground fires.

Fire severity changed the bacterial PLFA profile. High tree‐related fire severity can not only reduce rhizosphere‐ and mycorrhiza‐associated bacteria but also involve a loss of labile litter inputs from trees and understory vegetation (Hart et al., 2005). On the other hand, several studies have indicated that fire severity mainly affects soil bacterial communities by altering the soil edaphic properties, especially pH (Adkins et al., 2020; Mikita‐Barbato et al., 2015; Xiang et al., 2014). In our study, partial loss of the organic horizon was associated with increasing pH along the soil‐related fire severity gradient, and the effects of fire severity on bacterial PLFAs may be due to the loss of organic matter, and other physical–chemical changes (e.g., accumulation of pyrogenic toxic compounds), or a combination of these factors. In the mineral soil, bacterial communities were not affected by fire severity, probably because the mineral soil is buffered against the detrimental effects of fire (Certini, 2005) and deeper refuges may constitute an important source of bacterial inoculum postfire.

Primary consumers (i.e., bacteria, fungi, and archaea) serve as the trophic “base” for the soil faunal community (Coleman et al., 2004), so cascading bottom‐up effects through the soil food web were expected to occur along the fire severity gradients (Gongalsky et al., 2021). Contrary to our third hypothesis, soil‐related fire severity had no clear influence on the abundance of any of the soil fauna groups investigated. However, we found that high tree‐related fire severity reduced the abundance and richness of Oribatida. Many Oribatida species are fungivorous (Walter & Proctor, 2013), and their decline may be due to loss of food sources, such as ectomycorrhizal fungal hyphae that were strongly affected by tree mortality (cf., e.g., Remén et al., 2010). Another possibility is that the loss of canopy cover exposed the soil surface to direct sunlight, which likely reduced soil moisture and altered soil temperature dynamics, creating a harsher habitat for drought sensitive Oribatida (Lindberg & Bengtsson, 2005) and potentially affecting the microbial communities (Hart et al., 2005; Santana et al., 2010; Sun et al., 2016). Many Collembola species are also fungivorous (Berg et al., 2004), but we found Collembola to be unresponsive to fire severity. Our findings are in line with previous work showing that effects of disturbances are often stronger and last longer for Oribatida than for Collembola (Lindberg et al., 2002; Lindberg & Bengtsson, 2005; Malmström et al., 2008), because Oribatida have lower mobility and a longer life cycle (Hågvar, 2010; Lindberg & Bengtsson, 2005; Malmström, 2012). In our system, a large amount of brown needles was deposited on the ground after the fire, which might have facilitated the recovery of surface dwelling Collembola species and species with traits for fast, active dispersal, independently of fire severity (Malmström, 2012; Pollierer & Scheu, 2017), as compositional changes of Collembola after fires may mainly be associated with the loss of the litter layer (Huebner et al., 2012).

Fungal feeding nematodes and plant root hair feeding nematodes (which are also facultative fungal feeders) were less abundant in the burned stands, corresponding to the loss of live roots and associated ectomycorrhizal fungi. Similarly, previous studies involving vegetation removal or tree girdling have shown a strong bottom‐up control of fungivorous nematodes by decreased fungal abundance (Fanin et al., 2019; Kudrin et al., 2021). However, nematodes were unresponsive to either of the fire severity gradients within the burnt stands in our study, potentially because nematode communities were also influenced by other factors, such as understory vegetation, soil characteristics, and predation intensity (Magnusson, 1983).

Boreal forests are currently affected by anthropogenic global warming (IPPC, 2021). As a consequence, their wildfire regimes are forecasted to change in the future, with an increase in fire frequency, size, and severity (Baltzer et al., 2021; Burrell et al., 2022). Eurasian boreal forests are, in general, typically disturbed by frequent, low‐severity ground fires (De Groot et al., 2013; Rogers et al., 2015). It has been suggested that such forests may be less well adapted to future fire regimes expected to be more severe and more often stand‐replacing (Miller & Safford, 2020) with important consequences for postfire conifer regeneration and in the long run the maintenance of conifer‐dominated forests (Baltzer et al., 2021; Barrett et al., 2020). The Västmanland burn represents an example of an unusually big fire following a long period of drought in Sweden, and one with substantial variability in fire severity. In our study, we specifically found that tree‐related fire severity induced stronger negative effects on survival and recruitment of understory vegetation and soil biota (especially those dependent on live roots) than did soil‐related fire severity. Our short‐term findings indicate that recovery of *P. sylvestris* stands might be retarded after stand‐replacing fires relative to fires that leave the canopy more intact. However, longer term observations are necessary in order to improve our understanding of the potential consequences of a changing fire regime. Our study included *P. sylvestris* forest stands that had been managed before the fire and provides improved understanding of how these resist and recover from fire in the short term. *P. sylvestris* is the most widely distributed pine species in the boreal forest, ranging from western Europe to eastern Siberia. Within the northern and western part of this region, even‐aged forestry is the most widely applied management model, and thus managed forests are likely to be affected by fire events more often in the future. While we found many of the short‐term responses of our stands to be similar to those previously reported from natural forests, our findings help fill a large knowledge gap in the fire literature on managed forests. As such, our study is the first to identify two different fire severity gradients resulting from fire in managed forests, and to provide new understanding of the consequences of these fire severity gradients across organism groups. Our findings may therefore also be of direct relevance for postfire management decisions aimed at biological conservation and/or production.

## AUTHOR CONTRIBUTIONS

**Leticia Pérez Izquierdo:** Data curation (lead); formal analysis (lead); writing – original draft (equal); writing – review and editing (equal). **Jan Bengtsson:** Conceptualization (equal); investigation (equal); methodology (equal); writing – review and editing (equal). **Karina E. Clemmensen:** Conceptualization (equal); investigation (equal); methodology (equal); writing – review and editing (equal). **Gustaf Granath:** Conceptualization (equal); investigation (equal); methodology (equal); software (supporting); writing – review and editing (equal). **Michael J. Gundale:** Conceptualization (equal); investigation (equal); methodology (equal); writing – review and editing (equal). **Theresa S. Ibáñez:** Investigation (equal); writing – review and editing (supporting). **Bjorn D. Lindahl:** Conceptualization (equal); investigation (equal); methodology (equal); supervision (equal); writing – review and editing (equal). **Joachim Strengbom:** Conceptualization (equal); investigation (equal); methodology (equal); writing – review and editing (equal). **Astrid Taylor:** Investigation (equal); methodology (equal); writing – review and editing (supporting). **Maria Viketoft:** Investigation (equal); methodology (equal); writing – review and editing (supporting). **David A. Wardle:** Conceptualization (equal); investigation (equal); methodology (equal); writing – review and editing (equal). **Marie‐Charlotte Nilsson:** Conceptualization (equal); funding acquisition (lead); investigation (equal); methodology (equal); project administration (lead); writing – original draft (equal); writing – review and editing (equal).

## CONFLICT OF INTEREST STATEMENT

The authors declare that they have no conflict of interest.

## Supporting information


Figures S1.–S4.

Tables S1.–S5.

## Data Availability

The data that support the findings of this study are openly available in Dryad with DOI 10.5061/dryad.41ns1rnhp. Fungal data were deposited in the Sequence Read Archive (https://www.ncbi.nlm.nih.gov/bioproject/PRJNA592420).
