# Supplementary material for: Fire severity as a key determinant of aboveground and belowground biological community recovery in managed even‐aged boreal forests
Source: Ecol Evol. 2023 May 17;13(5):e10086. doi: 10.1002/ece3.10086 (PMC10191780; doi:10.1002/ece3.10086)
Supplement: Supplementary file 1 — Figures S1.–S4. [file ECE3-13-e10086-s002.docx]

**Supplementary figures**

**Figure S1.** Photos taken from the Västmanland burn 2015-2018 representing control stands (no fire) (a), stands with low fire severity (and surviving Scots pine) (b-e), and stands with high fire severity (and dead Scots pine) (f-i).

**Figure S2**. Results of Pearson correlation coefficients (r) between different pairwise combinations of above and belowground variables affected by fire across the 25 burned stands. Significant values at P>0.05 are coloured. Values in blue indicate negative correlations and in red positive correlations. Increasing colour intensity corresponds to the magnitude of the correlation coefficient

**Figure S3**. Horn’s parallel analysis for the principal component analysis (PCA) shown in Figure 3. The variables included are pH, proportion of scorched stems, stem soot height, proportion of scorched crown, soil C and N stocks, C:N, organic topsoil depth, pine root biomass, deposited needle litter and tree mortality. Adjusted eigenvalues (Ev) higher than zero indicate the dimensions to retain.

**Figure S4**. Ordination Analysis of understory vegetation community composition displayed by NMDS. Vectors indicate the direction and magnitude of correlations among community assemblage and tree-related fire severity and soil-related fire severity indexes. Stress value shown at the bottom of the graph corresponds to two dimensions (k=2). Numbers refer to each of the burned forest stands (red dots) (see Table S1).
