# Supplementary material for: Fire severity as a key determinant of aboveground and belowground biological community recovery in managed even‐aged boreal forests
Source: Ecol Evol. 2023 May 17;13(5):e10086. doi: 10.1002/ece3.10086 (PMC10191780; doi:10.1002/ece3.10086)
Supplement: Supplementary file 2 — Tables S1.–S5. [file ECE3-13-e10086-s001.docx]

**Supplementary tables**

**Table S1**. Description and GPS coordinates of the forest stands used in this study.

| **Stand ID** | **Burned/Unburned**  **forests** | **Longitude** | **Latitude** |
| --- | --- | --- | --- |
| 1* | Unburned | 16.27806 | 59.83693 |
| 22 | Unburned | 16.27564 | 59.83756 |
| 23* | Unburned | 16.28715 | 59.87562 |
| 24* | Unburned | 16.19555 | 59.94034 |
| 26 | Unburned | 16.08365 | 59.88803 |
| 35* | Unburned | 16.14071 | 59.84594 |
| 36 | Unburned | 16.16095 | 59.83860 |
| 3* | Burned | 16.25487 | 59.85266 |
| 4* | Burned | 16.24805 | 59.84244 |
| 5* | Burned | 16.24594 | 59.84107 |
| 7 | Burned | 16.22853 | 59.84762 |
| 8 | Burned | 16.23097 | 59.85172 |
| 9 | Burned | 16.23584 | 59.85265 |
| 10* | Burned | 16.24412 | 59.84972 |
| 13* | Burned | 16.27091 | 59.84959 |
| 14 | Burned | 16.24114 | 59.83830 |
| 15* | Burned | 16.24828 | 59.85848 |
| 16* | Burned | 16.24539 | 59.86023 |
| 17 | Burned | 16.26183 | 59.85644 |
| 18 | Burned | 16.26633 | 59.85692 |
| 19* | Burned | 16.27457 | 59.86310 |
| 20* | Burned | 16.27800 | 59.86405 |
| 21* | Burned | 16.28078 | 59.86648 |
| 25* | Burned | 16.19562 | 59.93569 |
| 27* | Burned | 16.17420 | 59.92965 |
| 28 | Burned | 16.13378 | 59.91722 |
| 29* | Burned | 16.13577 | 59.91478 |
| 30* | Burned | 16.14141 | 59.89669 |
| 32 | Burned | 16.15082 | 59.88842 |
| 33 | Burned | 16.11873 | 59.89832 |
| 34 | Burned | 16.08040 | 59.90053 |
| 37* | Burned | 16.13075 | 59.87414 |

* stands for which soil animals were analysed.

**Table S2.** Stand characteristics (Mean ± SE) of unburned (n=7) and estimates of pre-fire stand characteristics reconstructed for burned (n=25) forest stands. na-= not applicable. Significant differences between stand characteristics of unburned and burned forest stands, as evaluated by General Linear Models (GLMs), are highlighted in bold. The level of significance is indicated by asterisks (*P<0.05;**P<0.01; *** P<0.001).

|  | **All trees** | ***Pinus sylvestris*** | ***Picea abies*** | ***Betula* *pendula* + *B.* *pubescens*** |
| --- | --- | --- | --- | --- |
| **(a) Unburned forests**  Stand age; 49 ± 2 years |  |  |  |  |
| Mean tree height (m) ^1^ | 18.3 ± 0.7 | 18.3 ± 0.6 | 18.4 ± 1.1 | 23 ± na- |
| Total no of trees ha^-1 2^ | 764 ± 80 | 573 ± 67 | 141 ± 41 | 50 ± 45 |
| Proportion of species (%) ^2^ | na- | 76.0 ± 6.2 | 18.9 ± 5.5 | 5.3 ± 4.4 |
| Diameter at breast height (cm) ^2^ | 19.0 ± 0.9 | 20.4 ± 0.4 | 15.9 ± 1.7 | 7.0 ± 0.9 |
| Tree basal area (m^2^ ha^-1^) ^2^ | 23.2 ± 2.3 | 19.4 ± 2.2 | 3.6 ± 1.2 | 0.2 ± 0.2 |
| Tree biomass (Mg ha^-1^) ^2,3^ | 128 ± 13 | 103 ± 12 | 24.4 ± 8.3 | 0.7 ± 0.6 |
| C in tree biomass (Mg ha^-1^) ^2,3^ | 64.3 ± 6.5 | 52.0 ± 5.9 | 12.0 ± 4.1 | 0.3 ± 0.3 |
|  |  |  |  |  |
| **(b) Burned forests**  Stand age; 46 ± 1 years |  |  |  |  |
| Mean tree height (m) ^1^ | 17.6 ± 0.4 | 17.6 ± 0.4 | 20.8 ± 1.4 | 19.1 ± 0.6 |
| Total no of trees ha^-1 2^ | **998 ± 50*** | **852 ± 37***** | 120 ± 27 | 25.5 ± 8.0 |
| Proportion of species (%) ^2^ | na- | 86.72 ± 2.2 | 10.9 ± 2.1 | 2.4 ± 0.7 |
| Diameter at breast height (cm) ^2^ | **16.9 ± 0.4*** | **18.1 ± 0.5*** | 11.2 ± 1.8 | 10.6 ± 2.1 |
| Tree basal area (m^2^ ha^-1^) ^2^ | 24.2 ± 0.9 | 22.7 ± 0.9 | **1.2 ± 0.2***** | 0.3 ± 0.2 |
| Tree biomass (Mg ha^-1^) ^2,3^ | 124.6 ± 5.9 | 115.8 ± 5.4 | **7.3 ± 1.7***** | 1.5 ± 0.9 |
| C in tree biomass (Mg ha^-1^) ^2^**^,^**^3^ | 62.6 ± 2.9 | 58.2 ± 2.7 | **3.6 ± 0.8***** | 0.7 ± 0.4 |

Note: Tree data are based on observations of each tree in a 10m-radius plot within each stand. Pre-fire characteristics of the burned stands are reconstructed from the total number of standing trees and post-fire logs present within the 10m-circular plot in each stand.

^1^ data are calculated from 20 standing live and/or dead (e.g. post-fire snags or logs) trees per plot.

^2^ data are calculated from all standing live and/or dead (e.g. post-fire snags or logs) trees per plot.

^3^ based on allometric biomass functions following Marklund (1988).

**Table S3**. Contribution of aboveground and belowground indicators of fire severity to the three first axes of a Principal Component Analyses (PCA). The variance explained by each of the three components or axes are indicated inside brackets.

|  | PC1 (42 %) | PC2 (25 %) | PC3 (12 %) |
| --- | --- | --- | --- |
| Humus depth | 0.16211 | **-0.80766** | -0.25095 |
| C:N | 0.25568 | **-0.67748** | -0.33889 |
| C stocks | 0.28811 | **-0.85909** | 0.38581 |
| N stocks | 0.24923 | **-0.59948** | **0.69691** |
| pH | -0.28563 | **0.50486** | **0.54342** |
| Deposited litter | **-0.69475** | -0.18928 | 0.19621 |
| Crown scorched | **0.92503** | 0.05041 | 0.05670 |
| Pine roots | **-0.89578** | -0.22225 | 0.21097 |
| Tree mortality | **0.94245** | 0.09555 | 0.02359 |
| Stem soot height | **0.93197** | 0.21138 | -0.00352 |
| Stem scorched | **0.61027** | 0.29658 | 0.41228 |

**Table S4**. Post-fire characteristics of the 25 burned stands (Mean ± SE). na-= not applicable, because not enough data. Measures for living trees are presented outside the parentheses, and dead trees (including logs and snags) within parentheses.

|  | **All trees** | ***Pinus sylvestris*** | ***Picea abies*** | ***Betula pendula + B. pubescens*** |
| --- | --- | --- | --- | --- |
| Total no of living trees ha^-1^ | 303 ± 79 | 289 ± 75 | 1.3 ± 1.3 | 12.7 ± 5.5 |
| Total no of post-fire snags ha^-1^ | 570 ± 75 | 472 ± 72 | 88 ± 23 | 10.8 ± 4.8 |
| Total no of post-fire logs ha^-1^ | 127 ± 23 | 94 ± 22 | 30.6 ± 8.9 | 2.5 ± 1.8 |
| Diameter at breast height (cm) | 18.4 ± 1.3 (14.7 ± 0.8) | 20.1 ± 0.9 (16.0 ± 0.7) | 13.9 ± na- (11.2 ± 1.8) | 8.4 ± 2.0 (10.3 ± 2.8) |
| Tree basal area of trees (m^2^ ha^-1^) | 8.9 ± 2.2 (15.3 ± 1.9) | 8.7 ± 2.2 (14.0 ± 1.9) | 0.01 ± 0.01 (1.1 ± 0.2) | 0.1 ± 0.1 (0.2 ± 0.2) |
| Biomass of trees (Mg ha^-1^) ^1^ | 46.1 ± 11.7 (74.6 ± 9.8) | 45.5 ± 11.6 (67.1 ± 9.5) | 0.1 ± 0.1 (6.4 ± 1.5) | 0.5 ± 0.4 (1.0 ± 0.8) |
| C in biomass of trees (Mg ha^-1^) ^1^ | 23.2 ± 5.9 (37.4 ± 4.9) | 22.9 ± 5.8 (33.7 ± 4.8) | 0.1 ± 0.1 (3.2 ± 0.7) | 0.3 ± 0.2 (0.5 ± 0.4) |
| Tree mortality (%) | 72.0 ± 6.7 | 67.6 ± 7.9 | 99.2 ± 0.8 | 55 ± 15 |
| Crown volume scorched (%) | 71.6 ± 6.9 | 70.8 ± 7.1 | 100 ± 0.0 | 100 ± na- |
| Proportion of tree stems scorched (%) | 8.3 ± 2.5 | 8.8 ± 2.5 | 2.6 ± 2.6 | 11 ± 11 |
| Stem soot height (m) | 4.0 ± 0.6 | 4.0 ± 0.6 | 4.9 ± 1.7 | 4.7 ± 4.1 |
| Fine root biomass (g m^-2^) ^2^ |  | 3.5 ± 1.3 |  | 14.6 ± 1.8 |
| Post-fire deposited needle litter (g m^-2^) ^3^ | 631 ± 66 |  |  |  |
| Amount of charred litter (g m^-2^) ^3^ | 118 ± 22 |  |  |  |
| Light transmission (%)^4^ | 87 ± 1 |  |  |  |

Note: Tree data are based on observations of each tree in a 10 m-radius plot within each stand. ^1^ based on allometric biomass functions following Marklund (1988). ^2^ Data from Pérez-Izquierdo et al 2019. ^3^ n=4; ≤ 4 mm. ^4^ In unburned forest stands light transmission is 66 %.

**Table S5.** Shannon’s index of diversity, species richness and frequency (mean ±SE) of the vascular and non-vascular plant species present in the understory of the burned and unburned forest stands used in this study. Values of t and P are derived from General Linear Models testing for differences between burned and unburned forest stands. Significant differences (P<0.05) are highlighted in bold.

|  | **Burned forests**  **(n=25)** | **Unburned forests (n=7)** | **t (P-value)^*^** |
| --- | --- | --- | --- |
| Shannon’s index of diversity (H) | 2.5 ± 0.0 | 2.3 ± 0.1 | **2.2 (0.038)** |
| Species richness (S) | 12.6 ± 0.5 | 11.3 ± 0.7 | 1.2 (0.231) |
| Species frequency^1^: |  |  |  |
| 1. Herbs | | | |
| *Chamaenerion angustifolium* | 31.3 ± 2.6 | 0.0 ± 0.0 | **11.8 (<0.001)** |
| *Epilobium montanum* | 0.12 ± 0.06 | 0.0 ± 0.0 | 1.1 (0.281) |
| *Epilobium* sp. | 0.06 ± 0.04 | 0.0 ± 0.0 | 0.7 (0.463) |
| *Linnaea borealis* | 0.08 ± 0.08 | 15.5 ± 8.6 | **-4.4 (<0.001)** |
| *Luzula pilosa* | 0.9 ± 0.3 | 0.6 ± 0.6 | 0.9 (0.396) |
| *Maianthemum bifolium* | 0.06 ± 0.04 | 0.0 ± 0.0 | 0.7 (0.463) |
| *Melampyrum pratense* | 0.0 ± 0.0 | 1.9 ± 1.2 | **-3.1 (0.005)** |
| *Melampyrum sylvaticum* | 0.08 ± 0.06 | 12.4 ± 5.9 | **-5.7(<0.001)** |
| *Rubus idaeus* | 1.0 ± 0.4 | 0.0 ± 0.0 | 1.6 (0.119) |
| *Rumex acetosella* | 0.2 ± 0.1 | 0.0 ± 0.0 | 0.5 (0.473) |
| *Senecio sylvaticus* | 9.7 ± 1.3 | 0.0 ± 0.0 | **6.2 (<0.001)** |
| *Taraxacum* sp. | 0.06 ± 0.04 | 0.0 ± 0.0 | 0.7 (0.463) |
| *Trientalis europaea* | 3.4 ± 1.7 | 3.8 ± 2.0 | -0.5 (0.615) |
| *Vicia sylvatica* | 0.2 ± 0.1 | 0.0 ± 0.0 | 0.7 (0.460) |
|  |  |  |  |
| 1. Ericaceous dwarf shrubs | | | |
| *Calluna vulgaris* | 2.3 ± 0.8 | 0.8 ± 0.7 | 0.9 (0.366) |
| *Vaccinium myrtillus* | 8.4 ± 1.8 | 66.4 ± 7.6 | **-8.2 (<0.001)** |
| *Vaccinium vitis-idaea* | 4.7 ± 1.3 | 45.1 ± 12.7 | **-5.9 (<0.001)** |
|  |  |  |  |
| 1. Grasses and sedges | | | |
| *Calamagrostis arundinacea* | 2.4 ± 1.1 | 2.1 ± 1.0 | -0.3 (0.732) |
| *Carex pilulifera* | 1.8 ± 0.7 | 0.0 ± 0.0 | 1.9 (0.073) |
| *Carex sp.* | 0.6 ± 0.5 | 0.0 ± 0.0 | 0.7 (0.475) |
| *Deschampsia flexuosa* | 17.4 ± 3.6 | 33.0 ± 9.5 | **-1.6 (<0.001)** |
|  |  |  |  |
| 1. Tree seedlings^2^ | | | |
| *Betula pendula* | 1.2 ± 0.4 | 0.0 ± 0.0 | **2.3 (0.010)** |
| *Betula pubescens* | 0.98 ± 0.2 | 0.14 ± 0.1 | 1.9 (0.059) |
| *Picea abies* | 0.04 ± 0.03 | 0.07 ± 0.07 | 0.49 (0.628) |
| *Pinus sylvestris* | 0.98 ± 0.2 | 0.14 ± 0.1 | **2.51 (0.018)** |
| *Populus tremula* | 2.1 ± 0.5 | 0.0 ± 0.0 | **3.5 (0.001)** |
| *Salix caprea* | 1.1 ± 0.4 | 0.0 ± 0.0 | **2.5 (0.018)** |
|  |  |  |  |
| 1. Ferns | | | |
| *Pteridium aquilinum* | 2.4 ± 1.3 | 0.2 ± 0.2 | 0.9 (0.382) |
|  |  |  |  |
| 1. Lichens | | | |
| *Cladonia rangiferina* | 0.0 ±0.0 | 3.8 ± 3.2 | **-2.7 (0.011)** |
|  |  |  |  |
| 1. Bryophytes | | | |
| *Ceratodon purpureus* | 21.2 ± 3.6 | 0.0 ± 0.0 | **5.1 (<0.001)** |
| *Dicranum polysetum* | 0.0 ± 0.0 | 11.4 ± 2.4 | **-19.9 (<0.001)** |
| *Dicranum scoparium* | 0.02 ± 0.02 | 0.6 ± 0.4 | **-2.5 (0.018)** |
| *Hylocomium splendens* | 0.0 ± 0.0 | 35.4 ± 8.8 | **-11.77 (<0.001)** |
| *Marchantia polymorpha* | 1.1 ± 0.6 | 0.0 ± 0.0 | 1.6 (0.132) |
| *Pohlia nutans* | 0.5 ± 0.2 | 0.0 ± 0.0 | 1.8 (0.089) |
| *Pleurozium schreberi* | 0.02 ± 0.02 | 73.0 ± 4.8 | **-56.9 (<0.001)** |
| *Polytrichum juniperum* | 6.1 ± 2.1 | 0.0 ± 0.0 | **2.5 (0.018)** |
| *Polytrichum pilliferum* | 0.4 ± 0.1 | 0.0 ± 0.0 | 2.0 (0.053) |
| *Polytrichum* sp. | 23.7 ± 2.7 | 0.6 ± 0.6 | **6.6 (<0.001)** |
| *Sphagnum girgensohnii* | 0.0 ± 0.0 | 0.4 ± 0.3 | -2.0 (0.057) |
|  |  |  |  |

^1^ average of 8 quadrants per 10m radius plot

^2^ newly established tree seedling from seeds
